# Reactor engineering for the enzymatic synthesis of 5-hydroxymethylfurfural stearate in a batch bioreactor and a packed bed flow bioreactor

**DOI:** 10.1186/s40643-026-01036-1

**Published:** 2026-03-25

**Authors:** Nadia Guajardo, Nicolás Gajardo-Parra, Esteban Cea-Klapp, Roberto Canales, Maria Elena Lienqueo, Georgina Sandoval

**Affiliations:** 1 Departamento de Ingeniería Química y Bioprocesos, Escuela de Ingeniería, Pontificia Universidad Católica de Chile, Avenida Vicuña Mackenna 4860, Macul, Santiago, Chile; 2 Centro de Biotecnología y Bioingeniería (CeBiB), Departamento de Ingeniería Química, Biotecnología y Materiales, Universidad de Chile, Beauchef 851, Santiago, Chile; 3 Industrial Biotechnology Unit, LIBBA Laboratory, Center for Research and Assistance in Technology and Design of the State of Jalisco, A.C. (CIATEJ), 44270 Guadalajara, Jalisco Mexico

**Keywords:** Lipases, Biobased solvents, Flow biocatalysis, 5-hydroxymethylfurfural, Enzymatic esterification

## Abstract

The enzymatic esterification of 5-hydroxymethylfurfural (HMF) with long-chain fatty acids offers a sustainable route for producing biolubricants and other high-value chemicals. This work evaluates the synthesis of 5-hidroxymethylfurfural stearate catalyzed by immobilized lipases in both batch and continuous packed-bed bioreactors, combining molecular dynamics (MD) simulations with experimental validation to identify suitable green solvents. Four solvents were tested: 2-methyl-3-buten-2-ol (2-MB), tert-butanol (TB), 2-methyltetrahydrofuran (2-MeTHF), and cyclopentyl methyl ether (CPME). MD simulations revealed that CPME increased hydrophobic surface exposure and flexibility near the catalytic site, favoring substrate accessibility. In preliminary experimental tests, CPME provided the highest conversion (50%). In batch bioreactor at 40 °C, 30 mM HMF and 250 mM stearic acid achieved 67% conversion with *Candida antarctica* lipase B (CALB), maintaining full activity (100%) over four reuse cycles. In continuous operation, using a single packed-bed bioreactor at 0.02 mL min⁻¹ yielded conversions above 50% (residence time ≈ 55 min), while connecting two packed-bed bioreactors in series increased conversion to over 90% and productivity to 0.094 h⁻¹, compared with 0.076 h⁻¹ for one column and 0.003 h⁻¹ in batch mode. Deviations from ideal plug flow were observed over time, attributed to substrate or product deposition and in-situ water formation shifting the reaction equilibrium. Overall, CPME proved to be an efficient and sustainable solvent for the enzymatic synthesis of 5-hydroxymethylfurfural stearate, demonstrating the feasibility of continuous operation and highlighting pathways for further optimization through improved immobilization or reactor design.

**Graphical Abstract:**

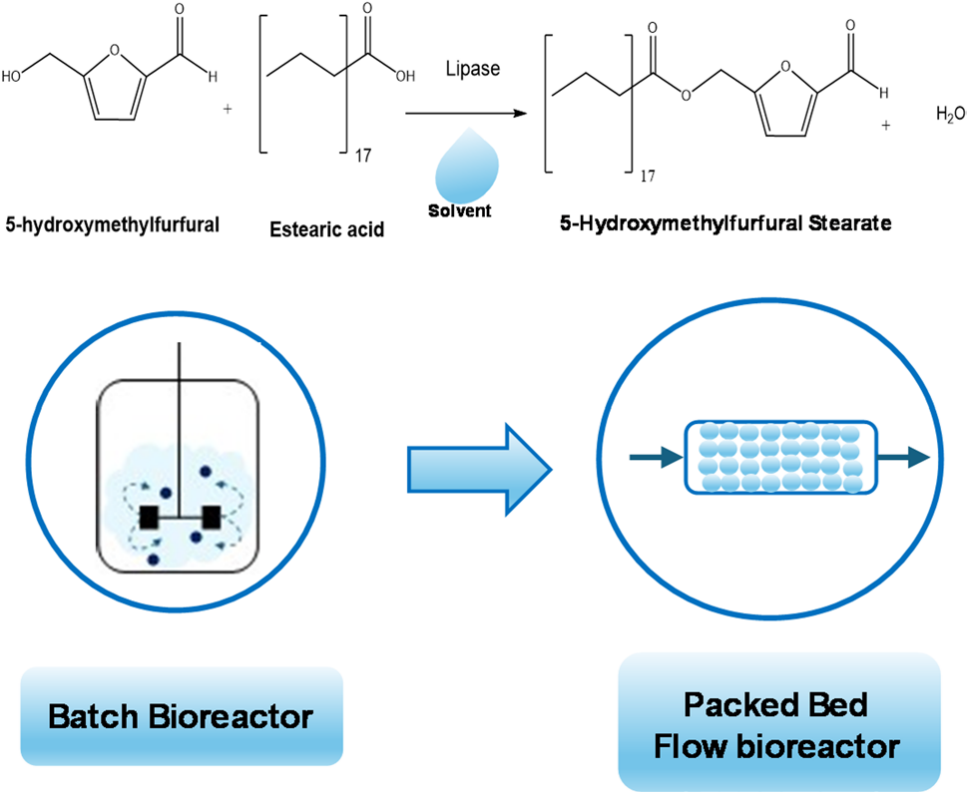

**Supplementary Information:**

The online version contains supplementary material available at 10.1186/s40643-026-01036-1.

## Introduction

The reduction in the availability of fossil fuels for energy generation and the manufacture of various compounds of commercial interest, such as plastics, solvents, and synthetic fibres, has intensified the search for renewable sources to ensure long-term sustainability (Ioannou et al. 2021; Sayed et al. 2021; Outeiriño et al. 2024; Bolaño Losada et al. 2025; Duque et al. 2025). One of the most promising approaches relies on the valorization of lignocellulosic biomass, particularly the residues generated during 2nd generation bioethanol production (Castro and Fernandes 2025). From these feedstocks, valuable furan-based platform molecules can be obtained, among which 5-hydroxymethylfurfural (HMF) stands out for its potential to be converted into high-value products such as bioplastics, liquid fuels, and esters (Balboa et al. 2022; Guo et al. 2022; Gómez-Meyer et al. 2023; Araya et al. 2024; Zuo et al. 2025).

Among the strategies used to functionalize HMF, esterification catalyzed by lipases offers a sustainable and versatile route (Uribe et al. 2023). These reactions are environmentally friendly and can generate products of commercial interest, such as fuel-blending agents, polymerization monomers to form biopolymers, natural surfactants for example lignosulfonates, fungicides, and biolubricants (Krystof et al. 2013; Qin et al. 2016a; Eid et al. 2021; Hu et al. 2021; Uribe et al. 2023; Ali et al. 2024a, b, c, 2026). Lipases belong to the group of hydrolase enzymes whose primary 0biological role is the hydrolysis of triglycerides (Mahfoudhi et al. 2022; Kumar et al. 2023; Vardar-Yel et al. 2024). The use of organic solvents in biocatalysis is crucial in some synthesis reactions, such as esterifications and transesterifications, as they reduce water by shifting the reaction equilibrium towards the products, allowing these reactions to proceed (Klibanov 1989, 2001; Cao et al. 2022). In addition to facilitating the displacement of water in the reaction medium, the solvent can dissolve substrates of different polarities, thereby providing a homogeneous reaction system (Villeneuve 2007). However, solvent selection is crucial in biocatalysis because it affects enzyme activity and stability. Therefore, it is essential to experimentally determine its effect on conversion and theoretically its impact on the enzyme, along with sustainability considerations, such as its origin from renewable sources (Stergiou et al. 2013; Remonatto et al. 2022; González and Guajardo 2023).

Owing to their versatility and broad applicability, lipases are widely used in both academic research and industrial processes (Agger and Zeuner 2022; Guajardo 2023). Furthermore, protein engineering and immobilization strategies have proven effective in enhancing their activity, stability, and selectivity, enabling the development of robust biocatalysts suitable for large-scale applications (Guajardo et al. 2021; Remonatto et al. 2022; Xie et al. 2022; Costa et al. 2023; Nie et al. 2025).

To better understand lipase behavior in nonaqueous media, researchers have employed molecular computational methods such as molecular dynamics (MD) simulations, which are powerful approaches for exploring solvent–enzyme interactions and the stabilization of enzymes at the molecular level (Shimizu and Smith 2004; Martínez 2022). For example, Mohtashami et al. (2019), explored the molecular mechanisms of enzyme tolerance to polar and nonpolar solvents via MD simulations of various enzymes such as laccases and lipases. In the specific case of enzyme esterification, (Duan et al. 2011) examined the effects of solvents on the positional selectivity of *Candida antarctica* lipase *B* (CALB) during the esterification of oleic acid with glycerol for 1,3-diolein synthesis.

The esterification of HMF with various compounds has been carried out since 1901 (Fenton and Gostling 1901), and over the past century, multiple strategies for its transformation have emerged, driven by the growing interest in biobased products. In most cases, strategies rely on the use of acids and bases, together with metal complexes, to increase the reaction rate (Mäki-Arvela et al. 2012; Weerathunga et al. 2021). In general, the esterification of HMF is challenging due to its high reactivity, since HMF can oligomerize and rehydrate to form levulinic acid, thereby decreasing yield and selectivity. In this context, biocatalysis offers a sustainable alternative owing to its mild operating conditions and high selectivity (Krystof et al. 2013; Domínguez de María and Guajardo 2017; Hu et al. 2018; González and Guajardo 2023).

There is still scarce available literature on esterification involving HMF. Reported enzymatic esterification with fatty acids has focused on chains shorter than 18 carbons (Krystof et al. 2013; Qin et al. 2016b; González and Guajardo 2023; Uribe et al. 2023). The incorporation of long-chain fatty acids, such as stearic acid, delivers new characteristics to the resulting esters, potentially making them attractive as biopesticides or biolubricants. However, these types of enzymatic reactions are challenging due to differences in substrate polarities, which typically require the use of a solvent to achieve a homogeneous reaction phase (Guajardo et al. 2017; Guajardo and Domínguez de María 2019; Guajardo and Schrebler 2024). This solvent must be carefully selected, as it should be sustainable, nonreactive with the substrates, and compatible with the biocatalyst. Motivated by these potential applications, this work aims to evaluate the esterification of HMF with stearic acid catalyzed by immobilized lipases, as illustrated in Figure S1. Stearic acid was used to provide greater viscosity to the biolubricant.

The esterification reaction was studied using four green solvents: 2-methyl-3-buten-2-ol (2-MB), tert-butanol (TB), 2-methyltetrahydrofuran (2-MeTHF), and cyclopentyl methyl ether (CPME). The selection of solvents was carried out using MD tools and experimental tests. The reactions were carried out in both batch and continuous packed-bed bioreactors.

Although there are studies in the literature on the esterification of HMF with fatty acids, this work incorporates a longer-chain unsaturated fatty acid (18 carbons) than those reported. Another novel aspect is the integration of MD tools to explain the interaction of the solvents and the enzyme, and the integration of flow biocatalysis to intensify the reaction in terms of productivity, heat transfer, and uniform product quality, thereby offering a promising route for enhancing the esterification process.

## Materials and methods

### Molecular dynamics simulations

MD simulations were performed via GROMACS 2022.1 to study the effects of different solvents on each enzyme (Abraham et al. 2015; Berendsen et al. 1995). Two lipases were considered: *Candida antarctica* lipase B (CALB, PDB ID: 1TCA) and *Thermomyces lanuginosus* lipase (TLL, PDB ID: 1DT3) (Uppenberg et al. 1994; Brzozowski et al. 2000). For simplification, the enzymes were simulated in their free (not immobilized) form to focus on solvation effects and avoid additional complexities associated with immobilization during the simulation (Duan et al. 2011). Enzyme topologies were generated via CHARMM-GUI (Jo et al. 2008; Lee et al. 2025), which employs the CHARMM36m force field at pH 7.0, excluding cofactors or ligands (Huang et al. 2016). Each enzyme was solvated in a periodic cubic box containing 3500 molecules of pure solvent. The solvent topologies for 2-MB, TB, 2-MeTHF and CPME were generated via CGenFF (Vanommeslaeghe and MacKerell 2012; Vanommeslaeghe et al. 2012).

After solvation, each system was subjected to energy minimization via the steepest descent algorithm for 50,000 steps. The Verlet cutoff scheme was used, with van der Waals interactions treated via a force-switch modifier that applied a switching function between 1.0 and 1.2 nm, while short-range electrostatic interactions were truncated at 1.2 nm. Long-range electrostatics were computed via the particle‒mesh Ewald (PME) method to ensure accurate treatment of Coulomb interactions beyond the cutoff distance (Darden et al. 1993).

The minimization step was followed by three equilibration stages, all of which used a 2 fs integration time step. The first stage consisted of 1 ns of NVT equilibration at 313.15 K, with protein heavy atoms restrained and the temperature maintained via a velocity rescaling thermostat (Bussi et al. 2007). This was followed by 5 ns NPT equilibration at 1 bar and 313.15 K via the Parrinello–Rahman barostat, during which restraints were maintained (Parrinello and Rahman 1981; Taylor et al. 2006). In the final stage, 1 ns of NPT equilibration without restraints was conducted to allow full relaxation of the protein within the solvent. Throughout all the equilibration phases, constraints were applied to all the bonds involving hydrogen atoms via the LINCS algorithm, and the center-of-mass motion was removed (Hess 2008). Subsequently, production simulations were carried out for 200 ns under NPT conditions (40 °C, 1 bar) without restraints. The coordinates were saved every 100 ps, and the energies were recorded every 2 ps. Periodic boundary conditions were applied in all directions. Post simulation analysis was performed via standard GROMACS to compute structural and dynamical properties, including the root mean square deviation (RMSD), root mean square fluctuation (RMSF), radius of gyration, and solvent-accessible surface area (SASA), on the basis solely of the production trajectories.

### Chemicals

HMF, 2-MB, TB, 2-MeTHF, CPME, and immobilized lipase from *Thermomyces lanuginosus* were purchased from Sigma‒Aldrich and used without modification. Stearic acid at 99% purity (Acros Organics, Geel, Belgium) was purchased from SAGULtda, Santiago, Chile. The immobilized lipase B from *Candida antarctica* (Novozyme 435^®^) was kindly donated by Blumos S.A. (Santiago, Chile). Formic acid and HPLC-grade methanol were purchased from Merck S.A., Santiago, Chile.

### Analytical method for the quantification of HMF

The reaction products were quantified via HPLC (JASCO LC- 4000 with a diode array detector) via a GL Sciences C_18_ HPLC column (100 × 4.6 mm I.D.) at 285 nm under the following conditions. The mobile phase comprised solutions A and B at a volume ratio of 85:15 (A/B), where A = water/formic acid (99.5/0.5) and B = methanol (100). The flow rate was 0.6 mL/min at 35 °C, and the retention time for HMF was approximately 5 min.

### Enzymatic esterification of HMF with stearic acid in a batch system

The enzymatic esterification of HMF with stearic acid in batch mode was carried out in a sealed reactor placed on a shaker at 165 rpm and 40 °C. The reaction medium consisted of 150 mg of biocatalyst and 2.5 mL of solvent containing HMF (15–30 mM) and stearic acid (250 mM) and was incubated for 72 h. The reaction was started by adding biocatalyst, and the reaction samples were periodically withdrawn for product analysis.

The conversion and productivity were calculated via Eqs. (1) and (2), respectively:

1 $$Conversion~\left( \% \right) = \left[ {\frac{{n_{{so}} - n_{s} }}{{n_{{s0}} }}} \right] \times 100 $$

where *n*_*S0*_ is the initial amount of substrate (HMF) and where *n*_*S*_ is the amount of substrate S at a given reaction time.

2 $$\mathrm{P}\mathrm{r}\mathrm{o}\mathrm{d}\mathrm{u}\mathrm{c}\mathrm{t}\mathrm{i}\mathrm{v}\mathrm{i}\mathrm{t}\mathrm{y}=\frac{M}{t\cdot{m}_{\mathrm{b}\mathrm{i}\mathrm{o}\mathrm{c}\mathrm{a}\mathrm{t}\mathrm{a}\mathrm{l}\mathrm{y}\mathrm{s}\mathrm{t}}}$$

Here, *M* is the mass of the product formed, *m*_biocatalyst_ is the mass of the biocatalyst, and *t* is the reaction time.

### Operational stability of the biocatalyst

The operational stability of the Novozym 435^®^ biocatalyst was determined in batch mode. The reaction medium consisted of 5 mL of solvent containing 30 mM HMF and 250 mM stearic acid, 300 mg of biocatalyst was used at 40 °C for 24 h. The biocatalyst was recycled by filtration and washed with CPME, and four reactions were carried out with fresh reaction medium. To ensure consistent reaction conditions across batches, the biocatalyst (mg)/ reaction medium volume (mL) ratio is adjusted.

Kinetic tests in bioreactors were performed with a reaction volume of 10 mL (CPME containing stearic acid and HMF) and 600 mg of biocatalyst. Approximately 100 µL of sample were extracted at different time intervals to quantify HMF levels using HPLC.

### Enzymatic esterification of HMF with stearic acid in a flow packed bed reactor

The enzymatic flow esterification of HMF with stearic acid in a packed-bed bioreactor to obtain 5-hydroxymethylfurfural stearate was carried out in a stainless-steel column (0.5 cm internal diameter and 14.4 cm long) manually packed with the biocatalyst, as shown in Figure S2. As in the batch bioreactor, the reaction medium was prepared by mixing HMF (15–30 mM) with stearic acid (250 mM) in 30 mL of CPME (volume of reaction medium). The reactions were carried out at different flow rates (0.02–0.5 mL/min, controlled by a peristaltic pump), and samples were taken at defined time intervals. The column temperature was maintained in a column oven. In the configuration with two columns in series, the second column has the same dimensions as the first column and contains an equal mass of biocatalyst.

The residence times were determined via Eq. (3):

3 $$ \tau = \frac{{ \in V_{t} }}{q} $$

where ∈ denotes the void fraction, *V*_*t*_ is the total bed volume and *q* is the substrate flow rate. The void fraction of the packed-bed bioreactor used in this work was calculated by using the average diameter of the biocatalyst particles, as determined by scanning electron microscopy, via the correlation proposed by (Benyahia and O’Neill 2005). The conversion rate and productivity were calculated according to Eqs. (1) and (2).

## Results and discussion

### Effect of solvent on protein dynamics

To obtain molecular insights into solvent–induced enzyme stabilization and flexibility, MD simulations were performed for CALB and TLL, with a focus on residue-specific motions in the presence of 2-MB, TB, 2-MeTHF, and CPME. A loss of structural integrity is observed for 2-MB, indicating the necessity of enzyme immobilization for its effective use as a solvent (Stepankova et al. 2013). Therefore, 2-MB is excluded from the following MD analysis.

**Fig. 1:**
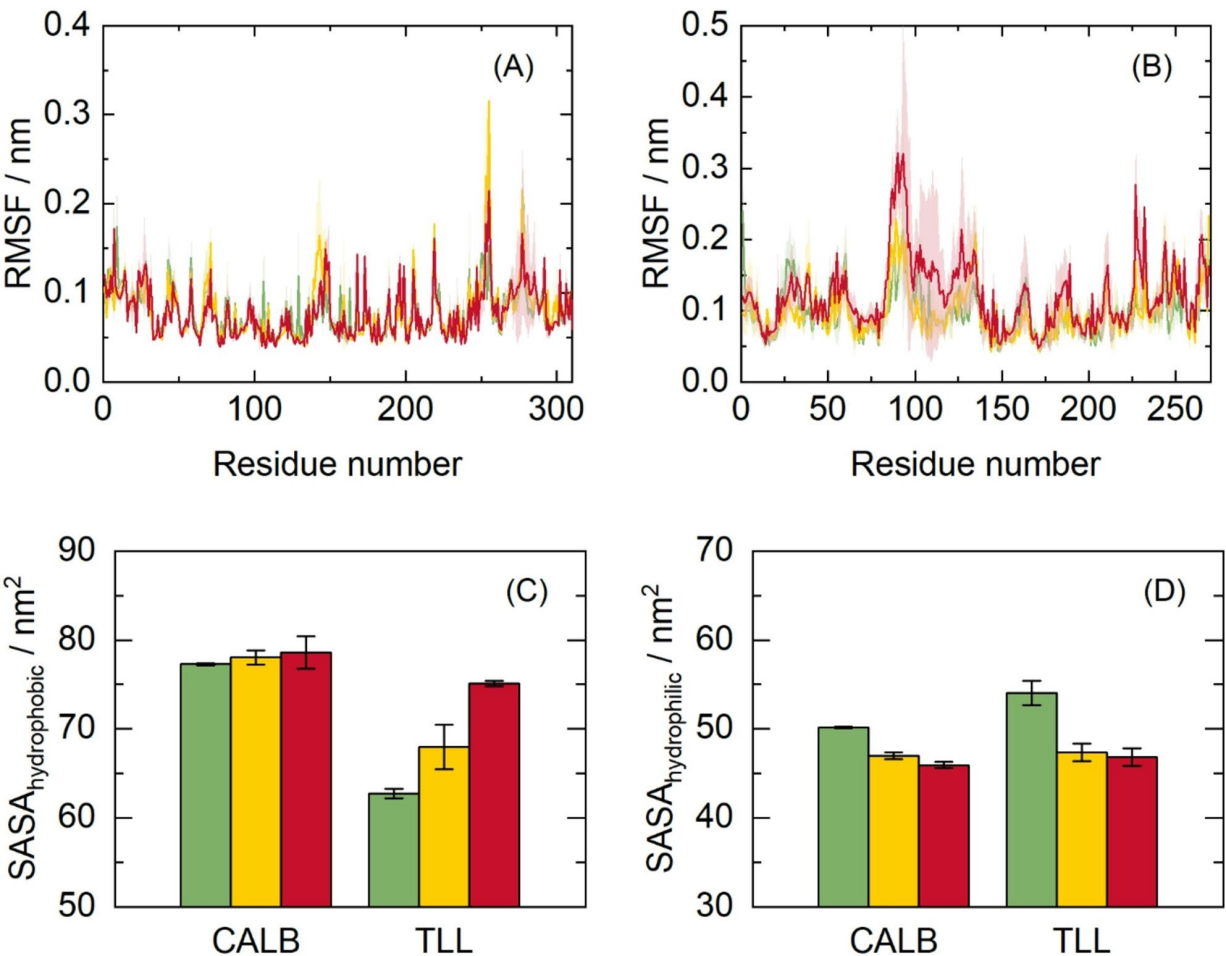
Protein alpha carbon RMSF of **A** CALB and **B** TLL in different solvents from MD simulations. CALB and TLL SASA for **C** hydrophobic and **D** hydrophilic groups in different solvents from MD simulations. Average values from the last 100 ns of simulations and standard errors computed from 3 independent runs at 40 °C and 1 bar. Solvents: TB (green), 2-MeTHF (yellow) and CPME (red)

The effect of the solvent environment on protein dynamics was monitored by calculating the RMSF of the Cα atoms for all residues, as shown in Fig. 1A for CALB and (B) for TLL. For CALB, fluctuations are dominated by the termini and exposed loops, but solvent-dependent perturbations around the catalytic triad (ASP187, HIS224 and SER105) are not observed (Galmés et al. 2020). Moreover, for TLL, there was a clearer coupling between the solvent, terminal regions, and the active site. CPME induces the greatest conformational plasticity among the solvents assessed. In CALB, the enhanced mobility localizes to the N- and C-terminal segments, whereas in TLL, the dominant fluctuations arise within the central scaffold that frames the substrate-binding cleft (Madsen et al. 2015). This pattern is supported by the elevated backbone RSMF, as shown in Fig. 1B. In TLL, greater core mobility accompanies a measurable decrease in tertiary packing density, indicating that CPME weakens hydrophobic-core cohesion and shifts the conformational ensemble toward more open, solvent-coupled states (Tong et al. 2016).

To assess how solvent media redistribute polar and nonpolar surface patches, SASA are computed and split into hydrophobic and hydrophilic contributions, as shown in Fig. 1C–D. In both enzymes, CPME shifts exposure toward hydrophobic residues while contracting the polar surface, a known effect in low-polarity solvents (Li et al. 2010).

For CALB, the hydrophobic SASA is nearly invariant across the three solvents, whereas the hydrophilic SASA decreases from TB (~ 50 nm²) to 2-MeTHF (~ 47 nm²) to CPME (~ 46 nm²), as shown in Fig. 1 (D). CPME evoked the strongest solvent-driven reorganization of the enzyme–solvent interface among the media examined. Figure 1C shows that in TLL, the nonpolar SASA increases substantially from TB (~ 62 nm²) to CPME (~ 76 nm²), whereas the polar SASA decreases from TB (~ 55 nm²) to CPME (~ 47 nm²), indicating a redistribution of surface exposure toward nonpolar patches in ether environments, with 2-MeTHF exhibiting an intermediate response. TB can hydrogen-bond with surface polar residues, stabilizing polar groups at the solvent interface and resulting in higher polar SASA. In contrast, CPME is less polar and lacks hydrogen-bond donation capability; thus, exposure of polar residues is less favorable, and the protein surface shifts toward lower polar SASA and higher nonpolar SASA. Collectively, these results indicate that ether media, particularly CPME, favor a protein surface enriched in nonpolar patches, a state that is expected to facilitate interactions with hydrophobic substrates and stabilize open, access-competent conformations under operationally relevant conditions (Rehm et al. 2001; Nazarian and Arab 2022).

### Selection of the solvent as a reaction medium

**Fig. 2:**
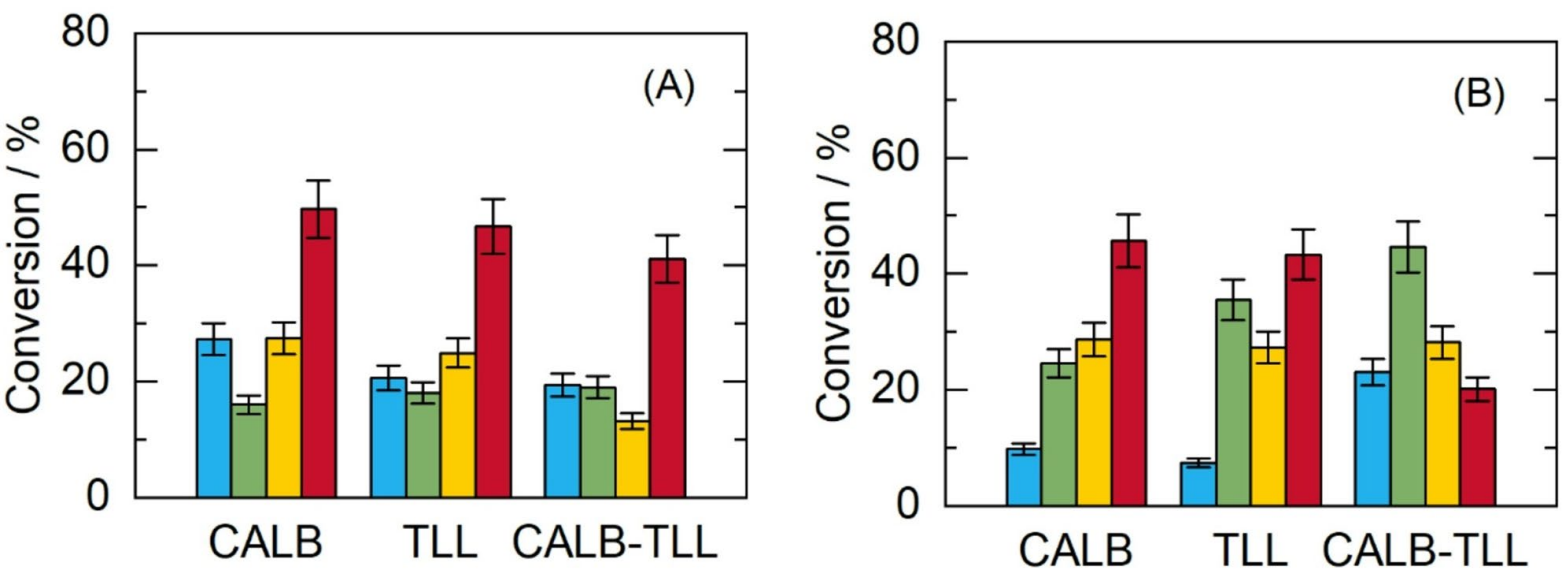
Conversion of HMF during enzymatic esterification at initial HMF concentrations of **A** 15 mM and **B** 30 mM, with stearic acid (250 mM), using CALB, TLL, or the combination CALB–TLL (75 mg-75 mg) at 50 °C. Reactions were carried out in 2.5 mL of reaction medium containing 150 mg biocatalyst, agitated at 165 rpm for 4 days. Solvents: 2-MB (blue), TB (green), 2-MeTHF (yellow), CPME (red)

The selection of the reaction medium is crucial for the reaction since it will improve the solubility of the substrates, and it must also favor enzymatic catalysis. An important aspect in selecting solvents is that they must be compounds that are not susceptible to esterification by the enzyme. For the selection, 2-MB, CPME, TB, and 2-MeTHF were used, and the results for different biocatalysts and substrate concentrations are detailed in Fig. 2. The best reaction performance was achieved with the CPME solvent. This behavior can be explained by the MD simulations, which showed that the CPME solvent imparts greater flexibility to the enzyme structure, exposing the nonpolar regions, which in turn facilitates the binding of the substrate to the enzyme’s active site.

In scientific literature, several examples have demonstrated the use of CPME as a reaction medium in enzymatic synthesis, which improves the enzyme’s activity, selectivity, and stability (Mine et al. 2005; Petrenz et al. 2015; Vernet et al. 2024). For example, Mine et al. (2005) reported that the CPME solvent modulated the activity and enantioselectivity of the *Pseudomonas cepacia* enzyme toward the *R*-enantiomer in the transesterification of 6-methyl-5-hepten-2-ol (racemic sulcatol: SUL). Comparing these results with ours, it can be inferred that, in our case, the biocatalyst was also more active in the presence of CPME, increasing the reaction rate and resulting in higher conversion. In the chemoenzymatic dynamic kinetic resolution of rac-benzoin, CPME also achieved a 1.6-fold increase in activity and a half-life up to 1.5 times longer than those of standard solvents such as toluene and 2-methyltetrahydrofuran (2-MeTHF), resulting in conversions ranging from 11% to 40% for *S*-benzoin (Petrenz et al. 2015). Regarding our results, the CPME solvent increased conversions 5 times compared to the 2-MB solvent, 2 times compared to the TB solvent, and 1.6 times compared to the 2-MeTHF solvent. Another successful example of the use of CPME as a reaction medium was the production of ε-caprolactone, which was catalyzed by immobilized cyclohexanone monooxygenases (CHMOs) and alcohol dehydrogenases (ADHs) in a cascade reaction. This method achieved 99.5% greater productivity than did a buffer solution, and the biocatalysts could be reused for 7 reaction cycles (Vernet et al. 2024). When comparing these results, mainly those on operational stability, with those obtained in our research, they showed a similar behavior, with the biocatalyst remaining active throughout the four reaction cycles. Based on these results, CPME will be used for reaction kinetics and operation in both batch and continuous packed-bed bioreactors.

### Reaction kinetics in a batch bioreactor

**Fig. 3:**
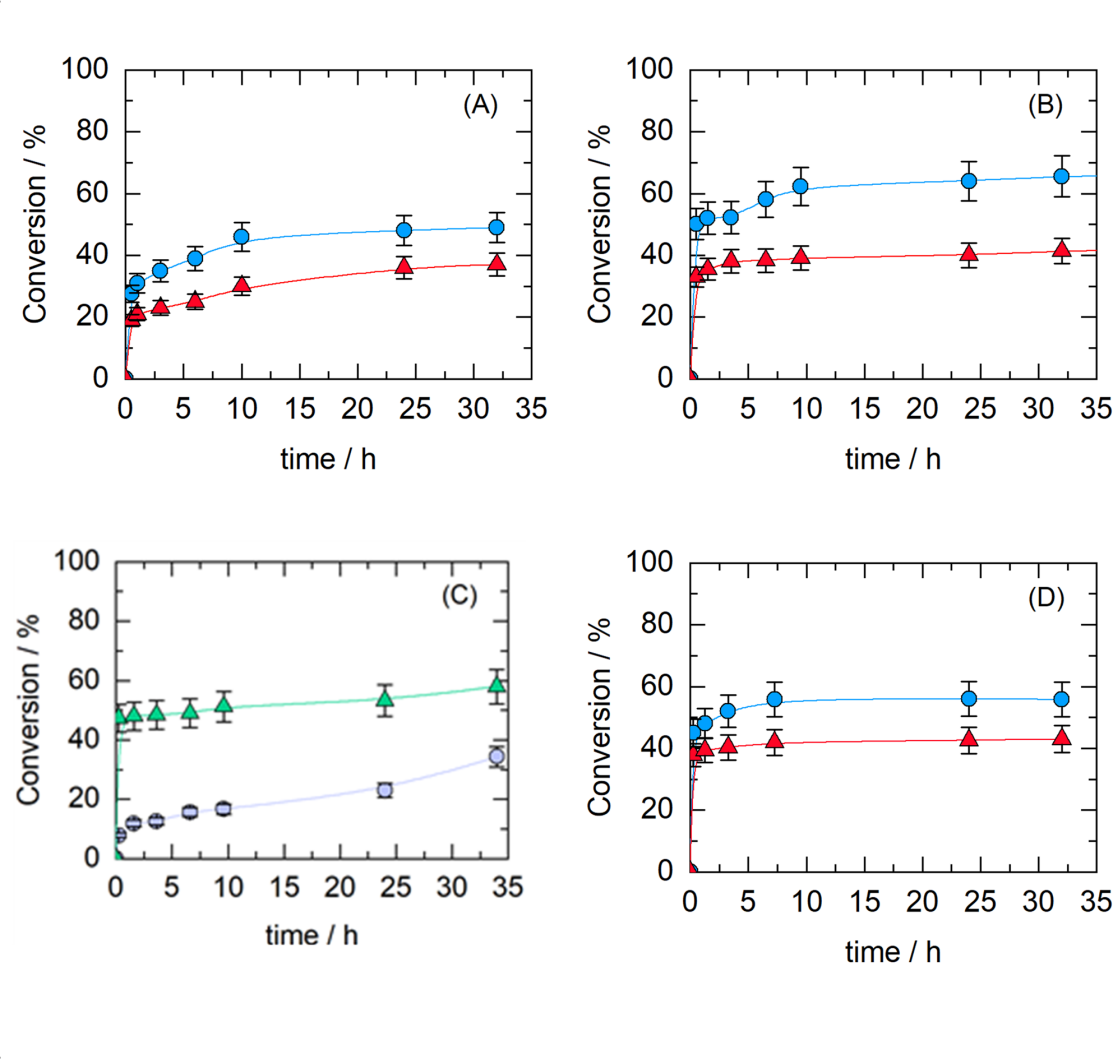
Conversion of HMF during enzymatic esterification in a batch bioreactor using 10 mL CPME as solvent, under different temperatures, substrate concentrations, and biocatalyst loadings. **A** 50 °C, 600 mg of CALB, 250 mM stearic acid, HMF 30 mM (blue) or 100 mM (red). **B** 40 °C, 600 mg biocatalyst, 250 mM stearic acid, HMF 30 mM (blue) or 100 mM (red). **C** 40 °C, 30 mM HMF with two conditions: 30 mM stearic acid + 600 mg of CALB (purple) or 250 mM stearic acid + 800 mg of CALB (green). **D** 40 °C, 600 mg of CALB with combined substrate concentrations: HMF 30 mM + stearic acid 30 mM (blue) or HMF 100 mM + stearic acid 400 mM (red)

Figure 3 shows the reaction kinetics in a batch bioreactor. The effects of the substrate concentration and reaction temperature on HMF conversion during enzymatic esterification with CALB were studied. Figure 3A, B shows that the reaction conversion is greater at 40 °C than at 50 °C. This behavior is noteworthy since enzymatic reaction rates are typically expected to increase with temperature according to the Arrhenius equation (Zou et al. 2025). However, in this case, the biocatalyst appears to be more stable at 40 °C, leading to greater overall conversion than at 50 °C. This behavior could be due to a synergistic effect between temperature and the CPME solvent at 50 °C.

To determine the effect of substrate concentration, experiments were conducted with an excess of stearic acid (250 mM) and varying concentrations of HMF (30 mM and 100 mM). This concentration ratio was used because, at equimolar concentrations of HMF and stearic acid, a low conversion rate was obtained, as shown in Fig. 3C. The highest conversion rate (67%) was achieved at a concentration of 30 mM HMF. This behavior could be attributed to the hydrophilic nature of HMF, which makes it more difficult to dissolve in the solvent CPME.

With respect to biocatalyst loading, increasing the amount of biocatalyst from 600 to 800 mg did not result in an increase in conversion during the enzymatic reaction (Fig. 3C). In some enzymatic esterification reactions, increasing the biocatalyst loading leads to an increase in conversion (Pedro et al. 2025). However, in other enzymatic esterifications, as in the case of this study, increasing enzyme loading does not necessarily increase conversion, as was also observed in the study by (Lee et al. 2025).

### Operational stability of the biocatalyst

**Fig. 4:**
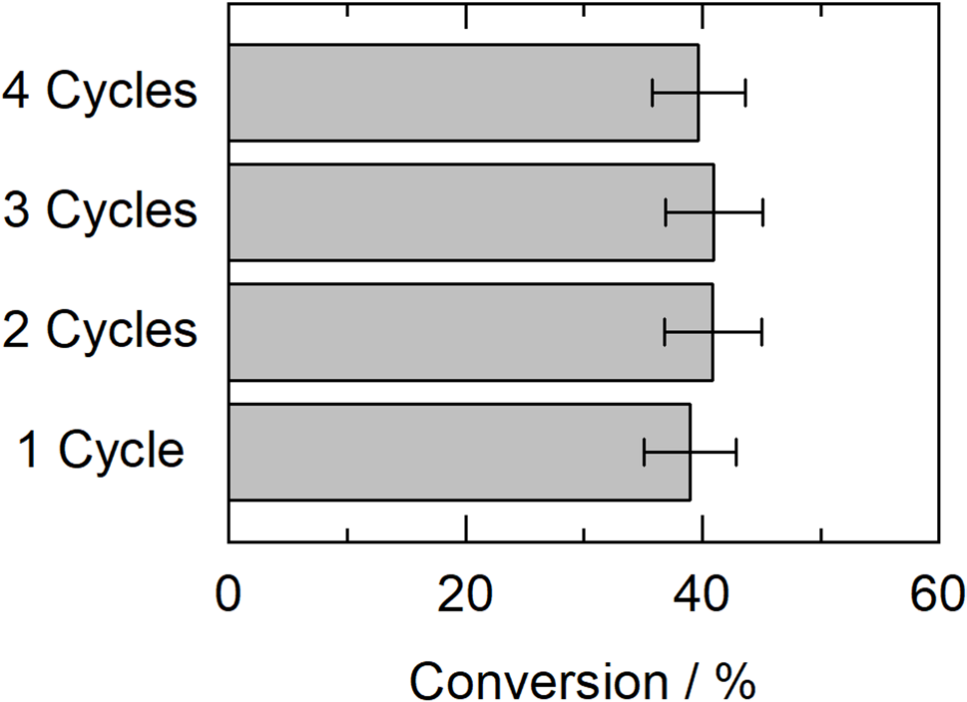
Operational stability of the biocatalyst in successive batch cycles. Reaction conditions: 5 mL CPME as solvent, 30 mM HMF, 300 mg CALB, 250 mM stearic acid, 40 °C, shaking at 165 rpm for 24 h

The evaluation of biocatalyst operational stability is critical to the successful development of biocatalytic processes. For this reason, the biocatalyst (CALB) was reused, and it was found to retain its initial activity after four reaction cycles, as shown in Fig. 4. This stable behavior of CALB has also been reported in other studies. For example, in the work of Valotta et al. (2023), a conversion rate of over 95% was achieved during 3 h of continuous reaction in the ring-opening polymerization of caprolactone catalyzed by CALB (Valotta et al. 2023).

Another example of the high stability of CALB in CPME was demonstrated in the synthesis of phenolic esters, such as 4-hydroxyphenethyl dodecanoate, in a continuous packed-bed bioreactor, where a conversion of 99% was achieved after 24 h of operation (Annunziata et al. 2022). The high stability of the biocatalyst, combined with the renewable nature of the solvent, makes CPME an attractive alternative as a reaction medium for enzymatic synthesis.

### Synthesis of 5-hydroxymethylfurfural stearate in a continuous packed-bed bioreactor

**Fig. 5:**
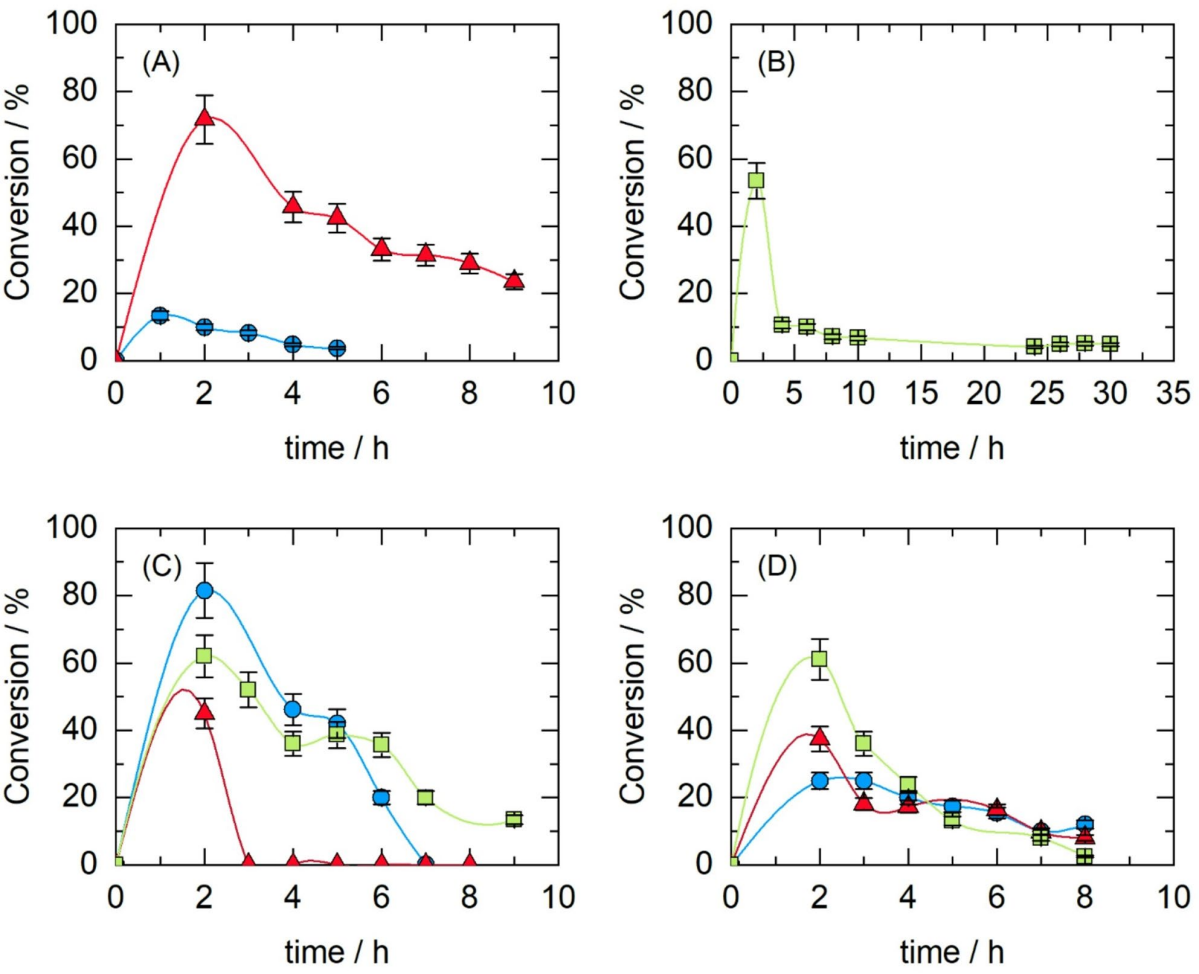
Conversion of HMF during enzymatic esterification in a continuous packed-bed bioreactor at 40 °C, under different flow rates, biocatalyst loadings, and substrate concentrations. **A** 30 mM HMF, 250 mM stearic acid, 30 mL CPME, 840 mg CALB, flow rates: 0.05 mL/min, τ = 240 min (blue) and 0.02 mL/min (red), τ = 600 min. **B** 30 mM HMF, 250 mM stearic acid, 30 mL CPME, 840 mg CALB, flow rate: 0.03 mL/min (green), τ = 400 min. **C** 30 mM HMF, three substrate combinations in 20 mL CPME, 840 mg CALB, flow rate 0.02 mL/min: 30 mM HMF + 100 mM stearic acid, τ = 400 min (blue), 50 mM HMF + 250 mM stearic acid, τ = 400 min (red), and 15 mM HMF + 250 mM stearic acid, τ = 400 min (green). **D** 20 mL TB, 250 mM stearic acid, flow rate 0.02 mL/min, with conditions: 30 mM HMF + 840 mg CALB, τ = 400 min (blue), 15 mM HMF + 840 mg CALB, τ = 400 min (red), and 30 mM HMF + 1270 mg TLL, τ = 400 min (green)

The operation of the continuous packed-bed bioreactor consisted of studying the effect of HMF conversion at different residence times and substrate concentrations. Figure 5A, B and C show that the best conversion was achieved with 30 mM HMF, 250 mM stearic acid, and a flow rate of 0.02 mL/min. Figure 5A, B and C show that there is a deviation from ideal behavior in continuous operation, which at first glance might suggest some biocatalyst inactivation. However, this hypothesis can be ruled out since the stability tests in sequential batch mode did not reveal any inactivation of the biocatalyst after four reaction cycles. Furthermore, the literature review did not indicate any decrease in activity in the presence of CPME. This behavior may be due to the deposition of stearic acid on the biocatalyst particles, which blocks the enzyme’s active site (Ognjanovic et al. 2009). This could occur because the laminar flow conditions in the system facilitate the deposition of acid onto the biocatalyst. Another possible cause is the generation of water within the continuous system, which shifts the reaction’s equilibrium (de Paula et al. 2021).

Many biodiesel-related works have reported solutions to reduce the binding of the reaction product to the biocatalyst support. One is changing the support to a more hydrophobic support, such as Accurel, or changing the solvent to a more polar one, such as TB, which has the opposite behavior from that of CPME in molecular dynamics simulations (Chen and Wu 2003; Al-Zuhair et al. 2011; Matte et al. 2016; Pollardo et al. 2018; Aguieiras et al. 2019). As observed in the preliminary experimental tests with TB (Fig. 2B), high conversions were also achieved. For this reason, it was decided to work continuously with this solvent. The results demonstrated that although the reaction conversion improved, the problem persisted (Fig. 5D). The alternatives that can be adopted in the future include developing an immobilization methodology using more hydrophobic support or solvents, another type of reactor or regenerating the column with some mixture of solvents (Chen and Wu 2003).

**Fig. 6:**
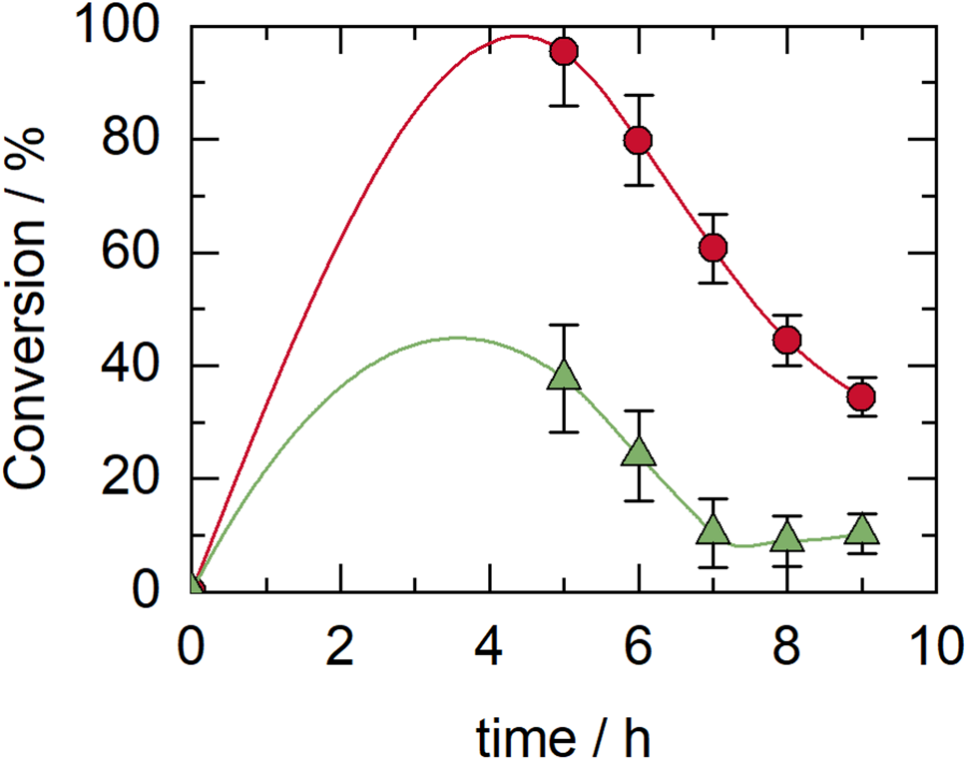
Conversion of HMF during enzymatic esterification using two continuous packed-bed bioreactors in series. Reaction conditions: 35 mL solvent (CPME, red; TB, green), 30 mM HMF, 250 mM stearic acid, 840 mg CALB, 0.02 mL/min and 40 °C

Experimental tests were carried out by incorporating a new continuous packed-bed bioreactor (Fig. S2B) with the same characteristics as the first one to evaluate the reaction conversion (Fig. 6). As shown in Fig. 6, a high conversion (90%) was observed in the first hours of the reaction, which decreased because the product adhered to the biocatalyst support, blocking the enzyme’s active sites and reducing its catalytic activity.

Table 1 presents a comparison of the reaction productivities under various operating conditions, indicating that the highest productivity is achieved in the continuous packed-bed bioreactor with two columns.

**Table 1:** Comparison of the productivity of different operating methods in the synthesis of 5-hydroxymethylfurfural stearate, considering the best results in each case

Operation mode	τ (min)	Productivity (g g^− 1^ h^− 1^)
Batch bioreactor	2880	0.003
One packed-bed bioreactor	55	0.076
Two packed-bed bioreactors in series	55	0.094

## Conclusions

The enzymatic synthesis of 5-hydroxymethylfurfural stearate, which operates in both batch and continuous packed-bed bioreactors, has been demonstrated for the first time. MD simulations and experimental conversion results in the presence of solvents indicated that CPME is the most suitable solvent for the reaction. In the batch bioreactor, when 30 mM HMF was used, a conversion of 67% was achieved. In the continuous packed-bed bioreactor, the highest conversions (above 50%) were obtained at a flow rate of 0.02 mL/min when a 30 mM HMF concentration was used. In the continuous flow system, a loss of reactor ideality was observed, possibly due to the deposition of stearic acid on the biocatalyst. Using two continuous packed-bed bioreactors in series, the highest conversion (over 90%) was achieved after 5 h of reaction. These results demonstrate that if the issue of bioreactor nonideality can be solved, a continuous flow system has significant industrial potential.

## Supplementary Information

Below is the link to the electronic supplementary material.

Supplementary Material 1.

Supplementary Material 2.

## Data Availability

Not applicable.
