# Supplementary material for: Reactor engineering for the enzymatic synthesis of 5-hydroxymethylfurfural stearate in a batch bioreactor and a packed bed flow bioreactor
Source: Bioresour Bioprocess. 2026 Mar 25;13(1):38. doi: 10.1186/s40643-026-01036-1 (PMC13018518; doi:10.1186/s40643-026-01036-1)
Supplement: Supplementary file 1 — Supplementary Material 1. [file 40643_2026_1036_MOESM1_ESM.docx]

**Reactor engineering for the enzymatic synthesis of 5-hydroxymethylfurfural stearate in a batch bioreactor and a packed bed flow bioreactor**

**Supplementary Information**

**Figure S1**. Enzymatic esterification of HMF with stearic acid in batch and flow packed-bed bioreactors.

**Figure S2.** Enzymatic synthesis of 5-hydroxymethylfurfural stearate in a packed bed flow reactor. A) Single column, B) Double column in series. 1) Packed bed flow bioreactor, 2) heated jacket, 3) peristaltic pump, 4) substrate feeding, 5) hot plate, 6) product.
