# Supplementary figures and images for: Reactor engineering for the enzymatic synthesis of 5-hydroxymethylfurfural stearate in a batch bioreactor and a packed bed flow bioreactor
Source: Bioresour Bioprocess. 2026 Mar 25;13(1):38. doi: 10.1186/s40643-026-01036-1 (PMC13018518; doi:10.1186/s40643-026-01036-1)

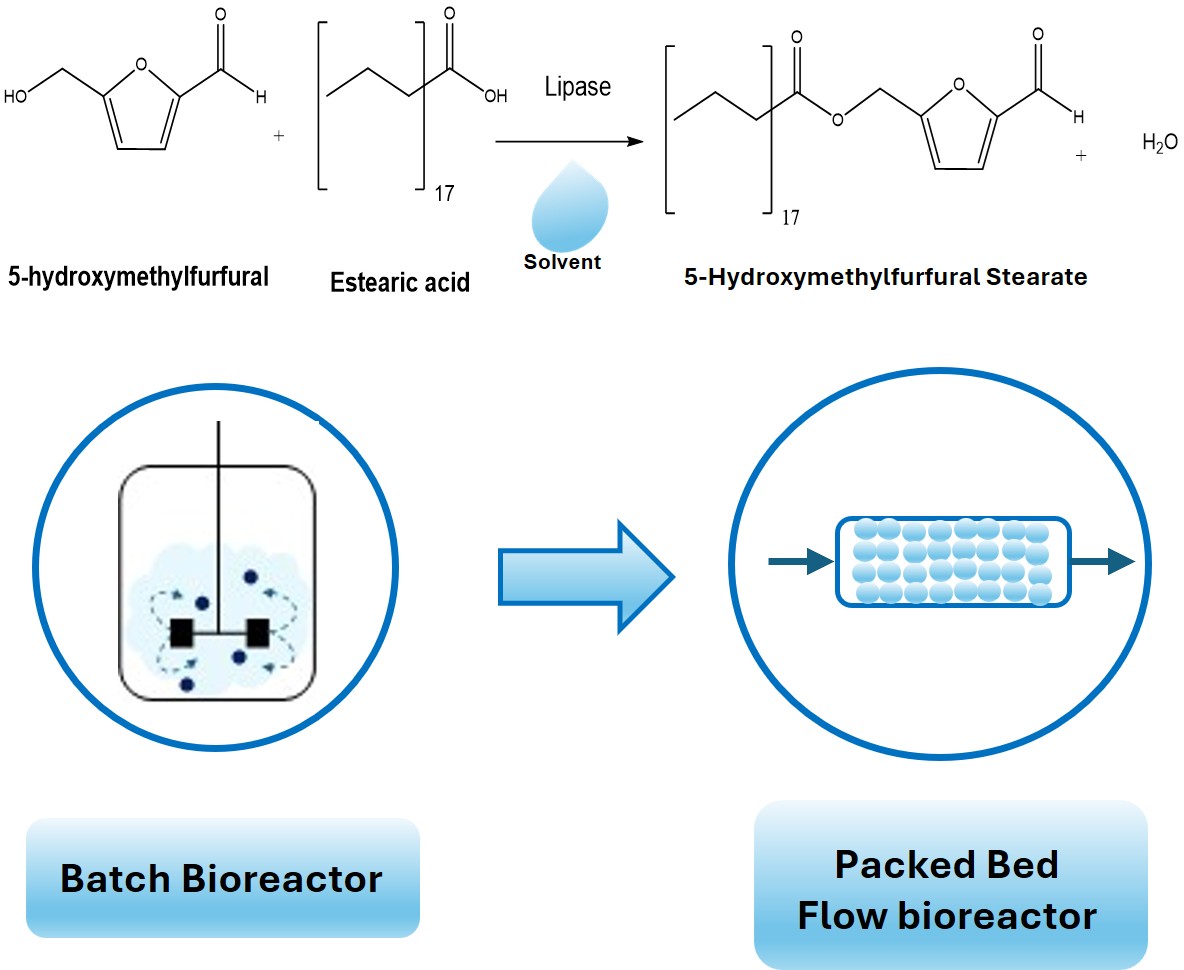

Supplement: Supplementary file 2 — Supplementary Material 2. [file 40643_2026_1036_MOESM2_ESM.jpg]
